# Beyond traditional vaccines: Semi-purified low-pathogenic avian influenza H9N2 virus-like particles and their promise for broiler immunity

**DOI:** 10.14202/vetworld.2024.2311-2321

**Published:** 2024-10-17

**Authors:** Muhammad Luqman, Sajjad Ur Rahman, Shafia Tehseen Gul, Muhammad Shahid Mahmood

**Affiliations:** 1 Institute of Microbiology, University of Agriculture, Faisalabad, Punjab, Pakistan; 2 Department of Pathology, Faculty of Veterinary Science, University of Agriculture, Faisalabad, Pakistan

**Keywords:** avian influenza virus, baculovirus expression system, H9N2, recombinant vaccine, virus-like particles

## Abstract

**Background and Aim:**

Avian influenza is a global threat to avian species, particularly in developing countries. Recombinant vaccines, including virus-like particles (VLPs), are promising strategies for preventing the spread of the disease. VLPs produced through the self-assembly of viral structural proteins without genomic material mimic native virions and are promising platforms for new vaccines. VLPs have been shown to elicit protective antibodies and are effective and safe vaccines against influenza. This study aimed to optimize the protocol for the production and characterization of H9N2 VLPs and their evaluation as a vaccine in broiler birds.

**Materials and Methods:**

Low-pathogenic influenza virus (LPAI) H9N2 was isolated and characterized through whole-genome sequencing, and a VLP-based vaccine for LPAI H9N2 was prepared using a baculovirus expression system. Codon-optimized hemagglutinin *(HA)*, neuraminidase *(NA)*, and *M1* were successfully cloned in pFastbac1 and expressed in SF9 cells. Proteins were characterized using sodium dodecyl-sulfate polyacrylamide gel electrophoresis (SDS-PAGE), western blotting, and electron microscopy after purification. Semi-purified proteins were tested as a vaccine in broiler chickens challenged with LPAI H9N2.

**Results:**

Recombinant Bacmid DNA from positive clones was extracted and confirmed using a polymerase chain reaction. The transfection showed cytopathic effects, and the proteins were confirmed through western blotting and SDS-PAGE, which showed the sizes of *HA* = 62–64 KD, *NA* = 52 KD, and *M1* = 25 KD. The shape and morphology were confirmed through transmission electron microscopy which revealed 100–150 nm size particles. As a result, the semi-purified VLPs (HA assay: 256) were tested as a vaccine for specific-pathogen free broiler birds; administered through subcutaneous and intranasal routes. The birds were challenged on the 28^th^ day after vaccination with the H9N2 strain, and the birds showed significant cross-reactivity with the H9N2 strain.

**Conclusion:**

The semi-purified VLP-based vaccine induced a significant immune response *in vivo*. This vaccine formulation has the potential to control avian influenza outbreaks in Pakistan’s poultry industry.

## Introduction

Avian influenza, commonly known as “bird flu,” primarily affects poultry, causing high mortality rates and economic losses [1, 2]. It is caused by low-pathogenic avian influenza (LPAI) and highly pathogenic (HPAI) viruses [3, 4]. HPAI strains, including H5N6 and H5N8, have caused outbreaks. This zoonotic threat has led to human infections and fatalities [5–7]. Between 2003 and 2022, H5N1 avian influenza caused 868 human cases and 457 deaths across 21 countries, and there is an ongoing global concern for both poultry and human health (World Health Organization, 2022 [8, 9]. Avian influenza viruses (AIVs) belong to the orthomyxoviridae family and are characterized by segmented genomes with eight single-stranded RNA segments. Hemagglutinin (*HA*), the most prevalent glycoprotein on the viral surface, allows the virus to attach to host cells by binding to terminal sialic acid residues, enabling the fusion of viral surfaces with receptors present on cellular membranes. Neuraminidase (*NA*), though less abundant, plays a crucial role in viral release by cleaving sialic acid from the viral membrane. AIVs exhibit 16 *HA* and 9 *NA* subtypes [3], with LPAI viruses commonly found in wild waterfowl. The H5 *HA* subtype is associated with zoonotic transmission concerns.

Rafique *et al*. [10] reported that vaccination is crucial to control avian influenza. Various technologies are used, including traditional inactivated AIVs; *HA* antigen or virus-like particles (VLPs) produced in insect cells through genetically engineered baculovirus, DNA-based vaccines, live virus vectors, and defective-replicating alphaviruses. These strategies help prevent outbreaks and protect poultry and human populations [11, 12]. Developing an effective, cross-protective, and safe *HA*-AIV vaccine for the poultry industry is crucial because of the challenges posed by time-consuming embryonated eggs/cell culture-based vaccines, inefficient vector-based/DNA vaccines, and continuous avian influenza strain mutations. VLP vaccines have gained popularity because they are self-assembled and non-infectious, providing increased safety. They resemble the original pathogen and stimulate both humoral and cellular immune responses [13, 14].

Baculovirus expression systems offer superior post-translational modifications. VLPs lack genetic material, mirror viral structures, and possess antigenic properties. Their morphology triggers robust immune responses, which are recognized by antigen-presenting cells. The repetitive VLP surface structure activates B cell receptors [15–17]. Pakistan, the 11^th^ largest poultry producer, faces the economic impact of AIV outbreaks (H7N3 and H9N2). Poor surveillance and biosecurity practices persist, and no recombinant vaccine production unit exists [10, 18].

This study aimed to optimize a VLP-based vaccine against the H9N2 strain using a baculovirus expression system, drawing on global evidence from vaccines such as Cervarix, Porcilis Pesti, Bayovac CSF E2, and FluBlok [13]. The baculovirus expression system can generate viral structural proteins that can spontaneously self-assemble into VLPs, making it a valuable tool for vaccine development and other biotechnology applications.

In this study, the *HA*, *NA*, and *M1* genes from H9N2 LPAI were selected with the highest similarity to all present Asian H9N2 LPAI strains, codon-optimized, and expressed and characterized using a baculovirus expression system, expressed and characterized. The semi-purified product was used for a preliminary trial on broiler birds in Pakistan. The birds were challenged with H9N2 LPAI and were monitored for VLP immunogenicity. The aim of the study was to optimize the protocol for the production of VLP vaccines in Pakistan.

## Materials and Methods

### Ethical approval

This study was approved by the Biosafety and Bioethical Committee of the Institute of Microbiology, University of Agriculture Faisalabad (IOM, UAF) and the Office of Research, Innovation, and the Commercialization University of Agriculture Faisalabad (3741/ORIC, UAF) ensuring compliance with ethical standards.

### Study period and location

The study was conducted from July to September 2022 at the animal house of the Institute of Microbiology, University of Agriculture, Faisalabad (IOM, UAF).

### Virus and cell culture

We previously characterized the AIV H9N2 strain (unpublished data) from domestic poultry in Pakistan. Briefly, the virus was isolated using 9-day-old specific-pathogen free (SPF) chicken embryonated eggs. The allantoic fluid was first confirmed through a hemagglutination test, followed by reverse transcriptase-polymerase chain reaction (RT-PCR) (Qiagen one-step RT-PCR, Germany). Final isolates were confirmed using next-generation sequencing (NGS) (UMGC, UMN, USA), and data were submitted to the National Center for Biotechnology Information (NCBI). The NGS-confirmed isolated strain was harvested using Madin-Darby Canine Kidney (MDCK) cells (Minimum essential media [MEM], 8% fetal bovine serum, 37°C with 5% CO_2_).

### Gene selection and cloning strategy

**Figure-1:**
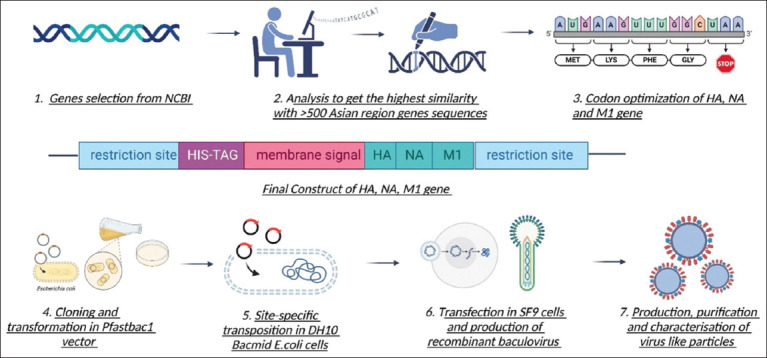
Complete workflow of target gene selection, codon optimization, cloning, expression, characterization, and virus-like particle purification.

The *HA*, *NA*, and *M1* gene sequences of the H9N2 AIV strain were obtained from NCBI. We compared all three sequences with already known Asian *HA*, *NA*, and *M1* (H9N2) to obtain the consensus sequence using BLAST. The final conserved sequence was selected from NCBI (MW767044.1, MW786666, and MH180452.1). Using the transmembrane prediction online tool (TMHMM 2.0), the *HA* transmembrane signal was replaced with the honeybee melittin signal to generate the *HA* protein expressed in the supernatant of Sf9 insect cells. Furthermore, the histidine marker (5’-8x-His) and restriction enzyme sites at 5’-Hind III and 3’-Sal I were added to the sequences before codon optimization (Figure-1). The sequence was codon optimized in accordance with Sf9 insect cells using Thermofisher and TwistBio, USA.

### Cloning of *HA, NA, and M1* genes into the pFastBac1 vector

**Figure-2:**
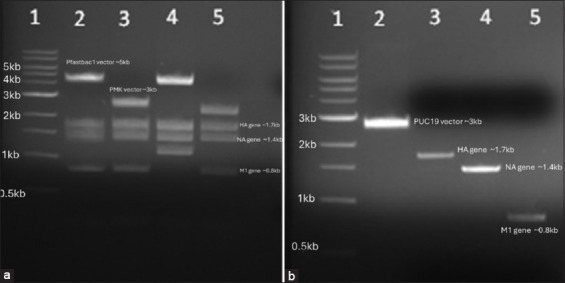
(a) Restriction enzyme digested final clones showing the digested vector, *HA*, *NA*, and *M1* genes (Lane 1 = 1 kb DNA ladder; Lanes 2 and 4 = Pfastbac1 with *HA*, *NA*, and *M1* genes; Lanes 3 and 5 = PMK vector with *HA*, *NA*, and *M1* genes;;). (b) Digested *HA*, *NA*, *M1*, and PUC19 vectors; lane 1 shows the 1-kb DNA ladder and lane 2 = Digested puc19 vector, lane 3 = *HA* gene, lane 4= *NA* gene, and lane 5 = *M1* gene.

*HA*, *NA*, and *M1* codon-optimized genes and the PUC19 cloning vector were digested with Hind III and Sal I (NEB) and ligated (T4 DNA ligase, NEB). The clones were transformed into one-short TOP10 *Escherichia coli* competent cells (C4040-10). The plasmid was extracted using a Qiagen minipump kit (27104, USA). The selected clones were confirmed through RE digestion and polymerase chain reaction (PCR) (Figures-2a and b). All three *HA*, *NA*, and *M1* genes were cloned separately and cloned together in the pFastBac I vector using the above-mentioned ligation and transformation strategy. Plasmids were extracted using a high-purity plasmid minipump kit (Invitrogen K2100-02, USA) and were confirmed through PCR and Sanger sequencing (UMGC, UMN, USA).

The confirmed *HA*-pFastBac-1, *NA*-pFastBac-1, *M1*-pFastBac-1, and *HANAM1*-pFastbac-1 plasmids were transformed in DH10. Bac *E. coli* cells were used to obtain recombinant Bacmid DNA. Positive-transformed *E. coli* cells were selected using LB agar plates containing kanamycin, gentamycin, tetracycline, blue gal, and IPTG. The triplet antibiotic with blue and white screening helped identify recombinant Bacmid DNA. In addition, it was confirmed using PCR. M13 primers and antibiotics were used according to the Bac-to-Bac system manual. Recombinant Bacmid DNA was extracted using a Hi Pure Maxi Prep Kit (Invitrogen, USA) (catalog No. K2100-02). All positive clones were preserved at –80^o^C in 50% glycerol solution.

### Transfection and expression in Sf9 cells

For the transfection and expression of recombinant *HA*, *NA*, and *M1* genes, Sf9 insect cells (Gibco Invitrogen-11496-015, USA) were used and maintained in ESF 921-1X, (96-001-01, USA) serum-free media. Both adherent and suspension cell cultures were maintained at 28°C in a shaking incubator. Sf9 cells were harvested and 3 × 10^5^ cells/mL per well were added to a 6-well plate and incubated for 2 h at 28°C without shaking to form a monolayer. We prepared 8 μL of Cellfectin II reagent (Gibco-10362-100) and 1 μg of each DNA sample and incubated them according to the guidelines provided by the Bac-to-Bac manual. The DNA transfection reagent complex was added drop by drop in 6-well plates after the removal of the media. The plate was incubated at 28°C for 5 h. Later, incubation was done as 3 mL of fresh media without antibiotics was added to each well after the removal of the DNA sample from the cell culture plate. Cytopathic effects (CPEs) were observed at 24-h intervals. After 72–96 h post-infection, P0 recombinant baculovirus was harvested, and plaque assay (Bac-to-Bac manual) was performed to check the titer. P1 and P2 were harvested to increase the stock and titer of recombinant baculovirus for protein expression.

For protein expression analysis, Sf9 cells were prepared in suspension cell culture (Erlenmeyer flask) and infected with recombinant baculovirus with a multiplicity of infection (MOI) of 5. After 96 h of incubation at 28°C, the entire mixture was centrifuged at 4000 × *g* for 45 min at 4°C. The supernatant and cell pellet were separately processed for protein purification and characterization.

### Protein purification

The cell pellet was lysed by sonication (Sonicator, S-4000 Misonix, Inc., USA) for 30 min at 20 amplitudes in ice with a 3/2 min start and pause cycle. The supernatant and lysed cells were ultracentrifuged at 3000 × g for 2 h at 4°C, 750 vacuum. For the purification of recombinant protein, a 20%–80% sucrose gradient with ultracentrifugation was used, and the pellet was collected from 40% and 60% of the sucrose layer. Semi-purified proteins were used for characterization through sodium dodecyl-sulfate polyacrylamide gel electrophoresis (SDS-PAGE), western blotting, and transmission electron microscopy (TEM).

### SDS-PAGE and Western blotting

Mini-PROTEAN Precast gel 4%–15% (456-8083, Bio-Rad, USA) with an unstained protein marker (12002064) was used for the initial screening of recombinant proteins from semi-purified lysed cell pellets and supernatants. For the final confirmation, primary monoclonal antibody hexa histidine epitope tags (6His-6402R, Abbexa Ltd., UK), influenza A *HA* H9N2 polyclonal antibody (PA5-81657, Thermofisher, USA), Influenza A *NA* H9N2 polyclonal antibody (catalog #. PA5-81732, Thermofisher), secondary antibody anti-rabbit IgG (Goat), HRP-labeled (catalog#. NEF812001, Thermofisher), and stained precision protein standard (1610375, Bio-Rad) were used. The transfer membrane was dipped for 60 s in Western lighting ultra-NEL 11200EA (equal amount of enhanced illuminant and oxidizing reagent). A Thermo Fisher MYCEL imager, USA was used to visualize the results under chemiluminescence and ultraviolet light using a UV transilluminator (Thermofisher).

### Transmission electron microscopy (TEM)

For the TEM analysis, a minimum of 100 μL of samples were taken in 600-μL tubes, and the iLAB facility of the University of Minnesota twin campus, St. Paul, Minnesota, USA, was used for the TEM. Copper 400-mesh formvar/carbon-coated grids were floated on 5 μL drops of the undiluted sample placed on a piece of Parafilm in a glass Petri dish. After 2 min, the grids were removed from the samples using forceps, the excess sample was swept away with filter paper, and then the grids were immediately floated on 10 μL drops of 2% aqueous uranyl acetate for 10 s. The grids were blotted and sample side-up on filter paper in a Petri dish to air dry. Grids were analyzed using a JEOL JEM-1400 Plus transmission 87 electron microscope (Japan) running at 60 kV. Images were captured with an XR16 Advanced Microscopy Techniques camera (Deben, UK) using the AMT Capture Engine software ver. 7.0.0.187 (https://www.amtimaging.com).

### Hemagglutination assay (HA assay)

The HA assay was performed using a previously described protocol by Pushko *et al*. [19]. Briefly, 2-fold serial dilutions of recombinant *HA* protein were prepared in 96-well plates and then 1% turkey erythrocytes were added and incubated for 45–60 min at 37°C. The *HA* titer was visually determined.

### Preliminary vaccine trial and virus challenge

For the trial, 50 μL of semi-purified cell culture supernatant (approximately 1 ug recombinant proteins with HA assay titer >256), including recombinant proteins, was inoculated through two routes (intranasally and subcutaneously) in SPF broiler chickens.

One-day-old 100 SPF broiler chickens were divided into four groups (n = 25 each group) and were used to determine the efficacy and immunogenicity of semi-purified recombinant proteins. Groups 1 and 2 were vaccinated at day 7 with semi-purified recombinant proteins subcutaneously and intranasally, respectively. Group 3 was vaccinated subcutaneously with a commercially available H9-killed vaccine as a positive control. Group 4 received intranasally administered phosphate-buffered saline (PBS) as a negative control. The booster dose was administered at 21 days in each group using the same route.

Feces and cloacal swab samples were collected according to the timeline. All groups were challenged with LPAI H9N2 (the same virus that was isolated and characterized) on the 28^th^ day, and weight, signs, and symptoms were routinely observed for the next 7 days. All birds were slaughtered by knife and bled on the 35^th^ day, and blood, lungs, trachea, and liver samples were collected for further analysis.

### Virus shedding and hemagglutination inhibition (HI) assays

The HI assay was performed according to the timeline to check the antibody titer. Serum was collected through centrifugation at 2000 × g for 3 min from blood samples collected from birds. In a 96-well round-bottom titration plate, a 2-fold serial dilution of serum was prepared in PBS. 4HA units of H9N2 virus were added and incubated at 37°C for 30 min. After incubation, 1% red blood cells were added and incubated again under the same parameters. Results were calculated visually. Virus shedding was determined by inoculating fecal and cloacal swabs from SPF chicken embryonated eggs. The titer was analyzed using the HA test.

### Statistical analysis

To create Kaplan–Meier survival curves, Prism 10.2.3 (GraphPad Co., San Diego, CA) was used. To compare the survival curves between the experimental groups, the Mantel–Cox log-rank test was utilized. One-way analysis of variance (ANOVA) was used to determine statistical differences in mean ± standard error of mean in average body weights in different treatment groups. 2-way ANOVA was used to determine significant differences in HI titers in different treatment groups. Tukey’s multiple comparisons test was performed to compare the means of HI titers in different treatment groups. All statistical analysis was carried out using a significance threshold of p < 0.05.

## Results

### Cloning and generation of recombinant Bacmid DNA

Codon-optimized *HA*, *NA*, and *M1* genes were successfully cloned in separate and in one cassette pFastbac1 cloning vector. Briefly, all three genes were initially ligated into the PUC19 vector and were preserved at –80°C after confirmation through PCR and Sanger sequencing. All three genes were separately cloned into a pFastbac1 vector and then cloned into a single cassette in the pFastbac1 vector. Positive-ligated clones were selected on ampicillin-containing LB agar, and final confirmation was performed using PCR and danger sequencing. The gel electrophoresis results show the successful confirmation of the cloning steps, highlighting positive bands and the integrity of the vectors and genes. The *HA*-PFastbac1, *NA*-pFastbac1, *M1*-pFastbac1, and HANAM1-pFastbac1 clones were used for the transformation of the *HA* gene into DH10-competent cells.

**Figure-3:**
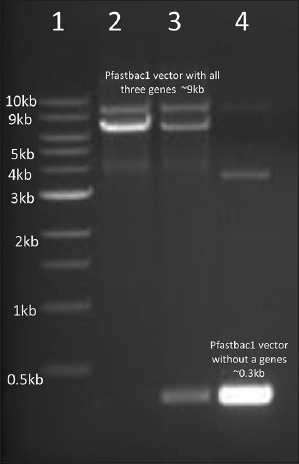
Confirmation of Recombinant Bacmid DNA (Lane 1 = 1 kb DNA ladder, lane 2 and 3 = Positive recombinant Bacmid DNA with M13 primers, lane 4 = Negative control Bacmid DNA).

The final step involved site-specific transposition of confirmed positive clones into DH10. Bac competent cells. Pure white and isolated colonies were selected, and Bacmid DNA was isolated using the Maxiprep HiPure kit (Invitrogen, USA). Confirmation of the recombinant acid DNA was achieved via PCR using gene-specific and M13 primers. The gel electrophoresis results shown in Figure-3 validated the successful generation of recombinant acid DNA, revealing positive bands with M13 primers and confirming the absence of bands in negative controls. This comprehensive experimental process involved meticulous steps in gene cloning, vector manipulation, and recombinant acid DNA generation, ensuring the successful integration and confirmation of target genes.

### Expression, purification, and characterization of VLPs

**Figure-4:**
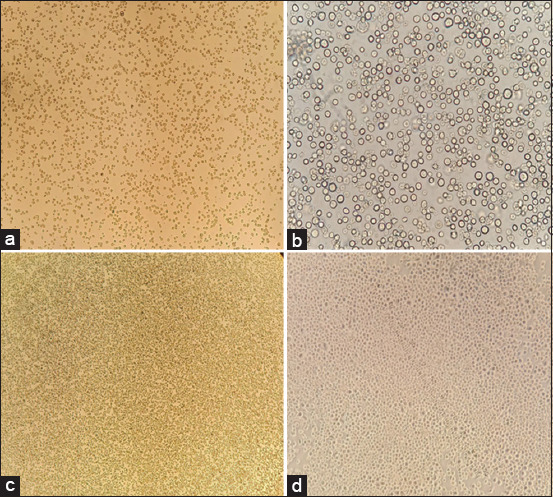
Monolayer of SF9 cells prepared at different time points. SF9 cells show confluency at different time periods, (a) after the addition of cells from the storage vial, most cells were suspended, (b) after 24 h the confluency of the cells at 10×, (c) cells confluency (80%–85%) after 48–60 h, (d) 100% confluency of the SF9 cells.

**Figure-5:**
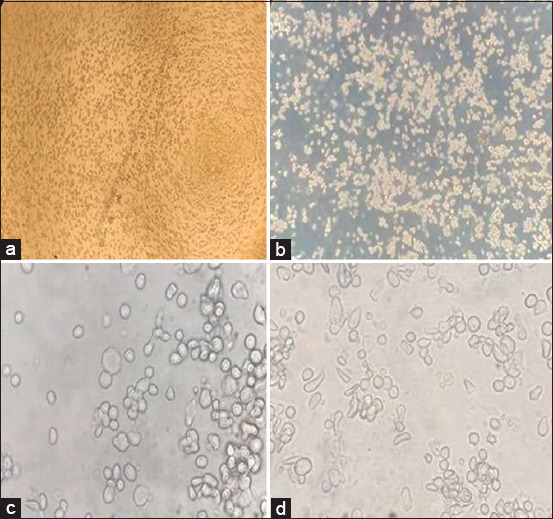
Recombinant Baculovirus shows cytopathic effects (CPEs) on SF9 cells. (a) CPEs of SF9 cells at 4×, (b) brightness of cells showing the number of infected cells at 10×, mostly cells detached after 48 h of infection, (c and d) CPEs of SF9 cells at 40×, showing elongated, multinucleated, and swollen cells.

In this experiment, Sf9 cells were cultured using both suspension and adherent methods in serum-free media to express VLPs. The growth and confluency of Sf9 cells were monitored over time, illustrating successful cell culture (Figure-4). Recombinant acid DNA was transfected into Sf9 cells using Cellfectin II reagent, leading to CPEs observed at various time intervals (Figure-5). Plaque assay confirmed the titer of the P1 recombinant baculovirus (3 × 10^6^ pfu). Following transfection, the P1 recombinant baculovirus was harvested to increase the titer, and Sf9 cells were infected (MOI: 0.1) in both monolayer and suspension cultures. Clear CPEs indicative of successful recombinant baculovirus infection (P2), were observed.

**Figure-6:**
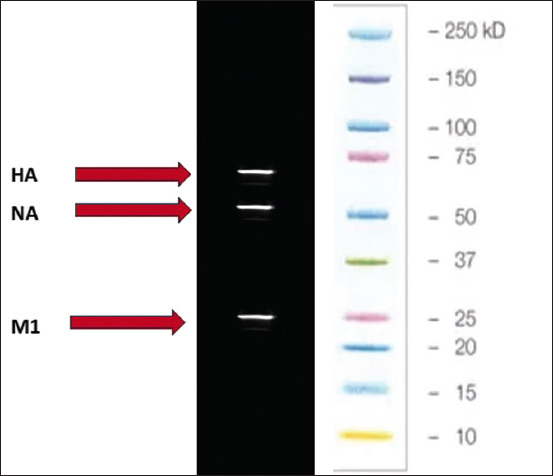
Hemagglutination assay, neuraminidase, and *M1* bands on western blotting.

**Figure-7:**
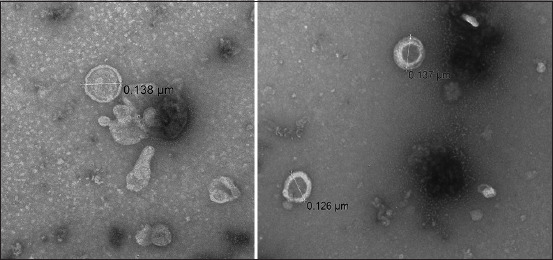
Transmission electron microscopy of the artificial intelligence virus-like particles.

**Figure-8:**
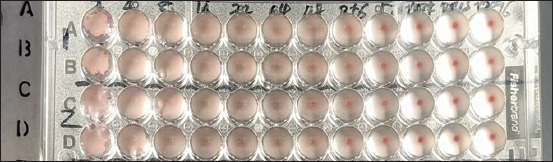
Hemagglutination assay titers of the artificial intelligence virus-like particles.

High-titer recombinant baculovirus was used to infect Sf9 cells with (MOI = 5) for the expression of recombinant proteins, after 72–96 h proteins were harvested from the supernatant and cells, followed by ultracentrifugation and ultrasonication. SDS and western blotting confirmed the presence of *HA*, *NA*, and *M1* in the purified samples Figure-6. The final step involved the observation of crude and purified products under TEM, which revealed VLPs with sizes ranging from 100 to 150 nm through negative staining Figure-7. After purification, *HA* was performed to confirm the titer of the recombinant *HA* protein, which showed an HAU of >256 per 50 μL (Figure-8). This comprehensive process demonstrates the successful expression, purification, and characterization of VLPs, which is essential for further studies and applications.

### Evaluation of VLPs as vaccines

**Figure-9:**
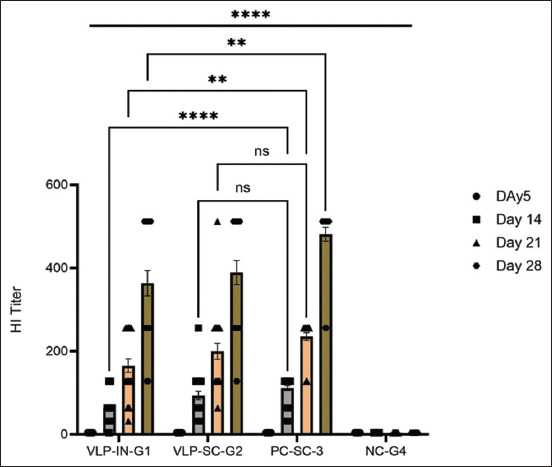
Statistical analysis of the antibody titer between groups. Data are presented as mean ± Scanning electron microscope with 25 biological replicates in each group. A 2-way analysis of variance showed a significant difference in the hemagglutination inhibition titer in different treatment groups. Tukey’s multiple comparison tests showed a significant difference in VLP-IN-G1 and VLP-SC-G2 from the negative group. A non-significant difference was observed between VLP-IN-G1 and VLP-SC-G2. A significant difference was observed between the VLP and PC groups. However, on days 14 and 21, VLP-SC-G2 showed a non-significant difference compared with PC-SC-3 (ns: Non-significant *0.0442, **0.0083, ****<0.001). VLP=Virus-like particle.

**Figure-10:**
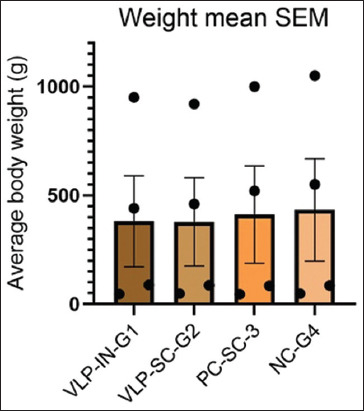
Mean body weight by scanning electron microscope (SEM) of all groups. The data are presented as the average body weight for each treatment group and showed as mean ± SEM at different time points (day 0, 5, 21, 28). One-way analysis of variance did not reveal a significant difference in body weight between groups.

**Figure-11:**
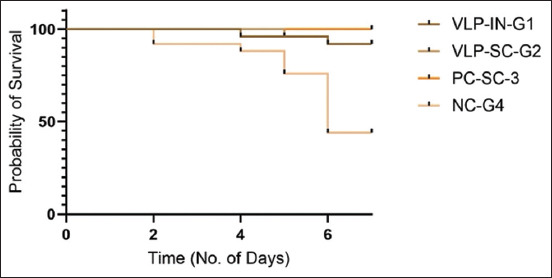
Survival curve was generated using Kaplan–Meier plot and Mantel–cox log-rank test showed a significant difference in the survival of animals in different groups (p < 0.0001). The survival curve was generated to assess the protective efficacy of the vaccine against viral challenge. Broiler birds were divided into four groups. The survival curve plots the percentage of surviving animals on the y-axis as a function of time on the x-axis. The vaccinated group’s curve showed a significant separation from the control group. The slope of the survival curve for the vaccinated groups was flatter, indicating a lower rate of death compared with the control group.

In the evaluation of VLPs as a vaccine, four groups (n = 25), each group of SPF chickens (n = 100) were administered primary and booster doses of VLPs through subcutaneous and intranasal routes. Group 1 was administered intranasally, group 2 was administered subcutaneously, group 3 served as a positive control with a commercial H9 vaccine administered subcutaneously, and group 4 was the negative control challenged with the virus without vaccination. On day 5, the VLP vaccine (>256 HAU) was administered without adjuvant, and the weight and antibody titers were measured. The first HI titer was measured on day 14, followed by a second booster dose on day 15 with the same titer. HI titers were measured on days 21 and 28. Group 2 (subcutaneous administration) showed a relatively higher GMT value compared with group 1 (intranasal administration), with significantly higher GMT values after the second dose. After the 28^th^ day, an H9N2 viral challenge (HA > 256) was administered, and the birds exhibited significant HI titers (Figure-9). The groups were closely observed for signs and symptoms for 7 days. Briefly, the results showed that there was no significant difference between chicken groups vaccinated with the VLPs-based AI vaccine (S/C and I/M) and birds vaccinated with the commercial AI vaccine. On the other hand, there was not any marked variation between the different groups in body weight, but at the 4^th^ week (immediately before the challenge), both the negative and positive control groups displayed a significant increase in body weight relative to the VLP-vaccinated groups (Figure-10). After the challenge, the VLP-vaccinated groups showed 100% morbidity in the first 4 days, with mild symptoms observed. Group 3 (positive control) showed 100% survival even after 6 days, with mild symptoms observed in some birds (Figure-11). Virus shedding was observed in all groups, with the highest shedding in the first group. The challenge used a low pathogenic AIV, resulting in 5-8% mortality. This comprehensive evaluation highlights the immunogenicity and protective efficacy of VLPs as a potential H9N2 influenza vaccine.

## Discussion

This study aimed to produce LPAI VLPs using a baculovirus expression system, optimize the protocol, and challenge broiler birds with LPAI H9N2. Optimization of the protocols can lead to the generation of VLP-based vaccines for other HPAI strains in infectious situations and for other viral diseases.

In this study, we investigated the immune response to a semi-purified VLP vaccine containing the *HA*, *NA*, and *M1* genes of LPAI H9N2 in broiler chickens in Pakistan. Previous study by Pushko *et al*. [19] using a baculovirus expression system to co-express these genes in Sf9 cells generated VLPs against H1N1, producing high titers of serum antibodies that prevented viral spread and cleared the virus from ferret lungs. VLPs provide protection by producing high levels of neutralizing antibodies [20], with intranasal delivery exhibiting higher immunogenicity and efficacy [21]. Maintaining the structural integrity and functional HA activity of VLPs was crucial for the development of protective immunity [22]. The inclusion of *M1* enhanced the CD8+ T-cell response, and *NA*’s slower antigenic changes compared with *HA* suggest it was a valuable vaccine target [21, 23–27]. Developing a vaccine based on the highly similar structural genes of AIV in Asia is a good strategy to induce a broadly protective immune response, and this study is the first in Pakistan to produce VLPs (recombinant proteins) using site-specific transposition in the baculovirus expression system.

In this study, consensus sequences of *HA*, *NA*, and *M1* were obtained by comparing all currently present sequences on NCBI based on Asian origin. LPAI H9N2 from domestic poultry in Pakistan was also isolated and characterized through NGS (accession numbers: OP107390, OP107755, OP108132, OP107397, OP107706, OP107707, OP1077043, OP107750, OP107752) and compared with these sequences to obtain the most similar sequence for vaccine production.

The reason for choosing the baculovirus expression system was that it is an effective expression tool for the large-scale assembly of recombinant proteins with high post-translational modifications. Furthermore, it provides a highly effective system for VLP development, boasting advantages such as high yields, rapid production, and the capacity to generate multivalent VLPs with essential post-translational modifications, ensuring proper self-assembly and release [13].

Various expression systems have been reported for AIV VLPs, including plant, mammalian, and insect cell expression systems [28–30]. However, the use of insect cells, particularly Sf9 cells, to generate a VLP influenza vaccine offers advantages over mammalian cells such as HEK293. Studies have shown that Sf9 cells produce significantly higher yields of VLPs (up to 35 times more) with higher HA titers and greater homogeneity than HEK293 cells. In addition, VLPs produced in insect cells exhibit a safety advantage as they are not likely to include human infections, and baculovirus vectors cannot reproduce in human cells [31]. Moreover, it has been observed that Sf9 insect cells do not incorporate sialic acids into N-glycans during post-translational modifications [32], which accounts for the efficient release of VLPs containing structural genes from insect cell surfaces [33] which makes this system an ideal system for generating VLPs.

For better secretions, expression, and translocation of recombinant proteins, we replaced *HA* peptide signaling with honey bee melittin signaling, which also indirectly stabilizes recombinant proteins [34]. In addition, the honeybee melittin signal peptide and a cleavable His tag in the pFastBac system increased the pure protein yield, simplified purification from serum-free culture supernatant, and may be applicable for expressing other viral or eukaryotic proteins [30, 35]. The polyhedrin promotor allows efficient, high-level expression of recombinant proteins. It also allows the insertion of genes in a frame with a melittin signal sequence for secretion, thereby permitting easy and efficient cloning of insert [36].

Two strategies were used for the expression of recombinant proteins: transfection of Sf9 cells with a single recombinant bacmid DNA containing all three genes and transfection of three recombinant bacmid DNA (*HA*, *NA*, and *M1* separately) into a single Sf9 plate. Interestingly, VLPs were confirmed by western blotting and TEM. In this study, data from one cassette (all three genes) is included. Different MOIs of the recombinant baculovirus were used for the protein expression such as (2, 3, 4, and 5), and were attempted by taking the supernatant at different time intervals (48, 72, 80, and 96 h) to optimize the maximum recombinant protein production and confirmed through SDS and western blotting. Hemagglutination activity further confirmed the functional stability and cRBC-binding activity of *HA* proteins on VLPs.

Recombinant proteins were confirmed by western blotting using anti-histidine and specific *HA* and *NA* antibodies. The same product was analyzed through TEM after negative staining that showed the 100–150 nm assembled particles. Western blotting and TEM confirmed that all three genes were expressed and assembled similarly to the influenza virus. The HA titers of the recombinant proteins also confirmed the proper structure and assembly of the structural proteins.

Studies have shown that purified VLP-based vaccines produced in the baculovirus expression system are highly purified products that can be used as vaccines in various animal models [37, 38]. Baculovirus is an insect virus that is not reported in mammals/birds as a pathogen [39]; therefore, instead of using purified products or purified products with adjuvants, we tried semi-purified cell culture supernatant as a vaccine in broiler chicken birds through the intranasal and subcutaneous routes. The intranasal route is the most important for poultry to ensure minimal human–bird interactions and bird stress. Our study demonstrated the efficacy of the semi-purified chicken product as a killed oil adjuvant commercial vaccine. Using chickens as a natural host and the main target for vaccination is a convenient, economical, and effective model. It also reflects the immune response and protection better than other animals, as studies have shown that single vaccination with chimeric bivalent VLPs induces strong immunity and reduced viral shedding [7, 13, 40]. These studies suggest that the chicken is a suitable and reliable model animal for H9N2 VLP challenge and vaccination.

A previous study by Jones *et al*. [41] demonstrated that even after two doses of vaccine containing 10μg of rHA protein, pure *HA* vaccination administered via the nasal route could not provide protection. Another study found that full protection might be induced by a two-component VLP vaccine (*M1* and *HA*), which has a much lower *HA* content (1 μg per dose) [33] than that in the above-mentioned study by Jones *et al*. [41]. Vaccination as a VLP elicits long-term immune responses and reactivation of B and T cells upon infection [42, 43]. Quan *et al*. [22] showed bone marrow-based differentiation of B cells into plasma cells, secretion of antibodies, and maintenance of robust protective immunity for extended periods, as demonstrated by effective protection against lethal virus challenges at 5 months post-vaccination. According to a recent study, administering a single dose of H6 VLPs with a commercial adjuvant resulted in a significantly increased level of antigen-specific antibody response compared with the other groups (measured on day 21). The study also found a long-term HI antibody response that lasted up to 4 months after vaccination [17]. In addition, the Quan group showed that intranasal immunization with PR8 VLPs provides complete protection against PR8 and WSN influenza strains, generating cross-reactive binding antibodies to demonstrate the ability of influenza VLPs as a versatile candidate vaccine. We excluded adjuvants from the semi-purified VLP vaccine based on previous studies showing enhanced immune responses and used a commercial adjuvant vaccine for comparison due to the lack of a recombinant H9N2 VLP vaccine on the market. Galarza *et al*. [33] have demonstrated protective effectiveness and immunogenicity against influenza virus in a murine model owing to the two components of the VLP vaccine (*HA* and *M1*). This study also demonstrated the protective effectiveness and immunogenicity of three components of VLP vaccination (H1, *M1*, and *NA*) in response to challenges with low-pathogen H9 influenza virus in chickens. Tukey’s multiple comparison tests (Figure-9) revealed significant differences in antibody titers between group 1 intranasal administration (VLP-IN-G1), group 2 subcutaneous administration (VLP-SC-G2) of vaccine, and the negative group, but no significant difference between VLP-IN-G1 and VLP-SC-G2. Significant differences were also observed between the VLP and positive control (PC) groups, except on days 14 and 21, where VLP-SC-G2 showed no significant differences compared with PC-SC-3. After the virus challenge, virus shedding (Supplementary data) on day 3 was higher in VLP-SC-G2 cells but significantly lower than that in the negative controls. All groups exhibited viral shedding on days 3 and 7 after the challenge. However, PC-SC-3 exhibited the lowest virus titer in virus shedding.

A survival curve was generated to evaluate the protective efficacy of the vaccine against viral challenge in broiler birds, which were divided into four groups. The survival curve depicted in Figure-11 plots the percentage of surviving animals on the y-axis as a function of time on the x-axis. The curve for the vaccinated groups showed a significant separation from the negative control group, with a flatter slope indicating a slower rate of mortality and a significantly higher probability of survival. However, compared with the positive controls, the vaccinated groups experienced mortality. We also observed a reduction in body weight in the vaccinated group, potentially due to several factors. First, the use of a semi-purified VLP vaccine may have caused stress and weight loss in the birds. Second, the vaccine trial and virus challenge were conducted in a non-Biosafety level 3 facility during the end of summer (rainy season), which may have further stressed the birds. We hypothesize that these mortality and weight loss may be attributed to the above-mentioned reasons. However, these VLPs induced an immune reaction that protected vaccinated chickens from the challenge of the H9 influenza virus compared with the negative control group. This implies that the *HA* and *NA* spikes exhibited neutralizing epitopes, indicating a structural conformation similar to that of wild-type *HA* spikes.

VLP vaccination has been proven to induce effective protection against influenza viruses induced by *HA*, *M1*, and *NA* surface proteins. This approach is advantageous because VLPs do not contain any genetic material from the influenza virus, making them a promising option for the development of prophylactic vaccines against potentially pandemic influenza viruses, including H5N1, H9N2, and H7N7. Furthermore, VLPs can be used to create monovalent and multivalent vaccines for a broad range of diseases by incorporating surface antigens of other pathogens. The safety, efficacy, and ease of delivery of this novel vaccine technology hold great promise for addressing serious public health issues. By pseudotyping and incorporating key antigens on the surface of the VLP, we can produce vaccines quickly and safely not only to fight influenza viruses but also to combat other emerging pathogens. Furthermore, multiple adjuvants can be combined with semi-purified products to assess immunogenicity in the future. Concerning the safety and efficacy of semi-purified VLPs, more work is required before applying this concept to the market.

## Conclusion

Our study shows that the VLP is an affordable and efficient alternative to conventional vaccine platforms. The study also highlighted the potency of the baculovirus expression system for creating viral proteins that are as native and conformational as their original analogs, resulting in a high capacity to induce host immunity and exhibit a protective response. In addition, our trial revealed that the VLP vaccine was just as effective when administered through the nasal route, even without mucosal adjuvant, as when administered subcutaneously. This finding increases the possibility of mass vaccine administration, which is highly recommended by vaccine producers.

## Data Availability

The supplementary data can be available from the corresponding author upon a reasonable request.

## Authors’ Contributions

ML, MSM, and SUR: Conceptualization, methodology. ML, STG, and MSM: Optimization. ML: Writing original draft. ML, MSM, and STG: Writing-reviewing and editing. STG and MSM: Project administration. All authors have read and approved the final manuscript.
